# Exploring the significance of fatty acid metabolism reprogramming in the pathogenesis of cancer and anticancer therapy

**DOI:** 10.1080/07853890.2024.2445774

**Published:** 2024-12-30

**Authors:** Jiaao Sun, Jiahua Liu, Feng Chen, Xiaoxi Wang, Guangzhen Wu

**Affiliations:** a Department of Urology, The First Affiliated Hospital of Dalian Medical University, Dalian, China; b Department of Clinical Laboratory Medicine, The First Affiliated Hospital of Dalian Medical University, Dalian, China

**Keywords:** Fatty acid metabolism, cancer, metabolic reprogramming, anticancer therapy, ferroptosis

## Abstract

One of the important markers that distinguish tumor cells from normal cells is metabolic reprogramming. Among them, the reprogramming of fatty acid metabolism not only supports the fuel supply for energy but also meets the needs of rapid biosynthesis, providing selective growth advantages for tumor cells to resist harsh microenvironments. Due to the crucial role of fatty acid metabolic reprogramming in tumor metabolism, its various functions in cancer have attracted more and more attention. In this review, we summarize the mechanisms of fatty acid metabolic reprogramming in tumor cells and the series of responses (such as active ferroptosis signaling), analyze the potential impacts brought by fatty acid metabolic reprogramming in various human cancers and focus on the significance of targeting fatty acid metabolic pathways and dietary control in cancer treatment.

## 1. Introduction

Fatty acids (FAs) are divided into saturated fatty acids (SFA), monounsaturated fatty acids (MUFAs) and polyunsaturated fatty acids (PUFAs), among which SFA is the main component of phospholipid and cholesterol; MUFAs can lower bad cholesterol and raise good ­cholesterol; among the PUFAs, arachidonic acid, homo-gamma-linolenic acid and eicosapentaenoic acid are involved in the synthesis of prostaglandins and participate in various subsequent biological processes. In addition, FAs can be further synthesized into complex lipid molecules, including phosphoglycerides (PG), sphingolipids, and triglycerides (TG). PG is the basic material of life, which can be divided into phosphatidylserine (PS), phosphatidylethanolamine (PE); TG is not only involved in energy storage, but also in the construction of cell structure; sphingolipid is an important component of biofilm structure [1,2].

Fundamentally, cancer is a disorder of cell growth and metabolism. It requires a significant amount of nucleic acids, proteins, lipids, and carbohydrates, and the energy produced by breaking them down. As a result, tumor cells often exhibit biosynthetic disturbances and bioenergy accumulation disturbances, which enable them to enhance raw materials for excessive proliferation and metastasis. Metabolic reprogramming is a key factor in the occurrence and development of cancer, including the reprogramming of glucose metabolism, FA metabolism, and amino acid metabolism. Among them, fatty acid oxidation (FAO) generates approximately twice the amount of energy compared to glucose, which is more advantageous for the occurrence of cancer [2,3]. Aberrantly active lipid biosynthesis and degradation are characteristics of cancer development, with uncontrolled FA metabolism playing a crucial role in the survival of tumor cells. This review summarizes the research progress on the reprogramming of FA metabolism in tumor cells, focusing on the effects of alterations in FAs metabolic pathways in various human cancers. Furthermore, it discusses the potential applications and future directions of targeting FAs metabolic reprogramming in anticancer therapies.

## 2. Unique synthesis, uptake, and oxidation of fatty acids in tumor cells

**Figure 1.**
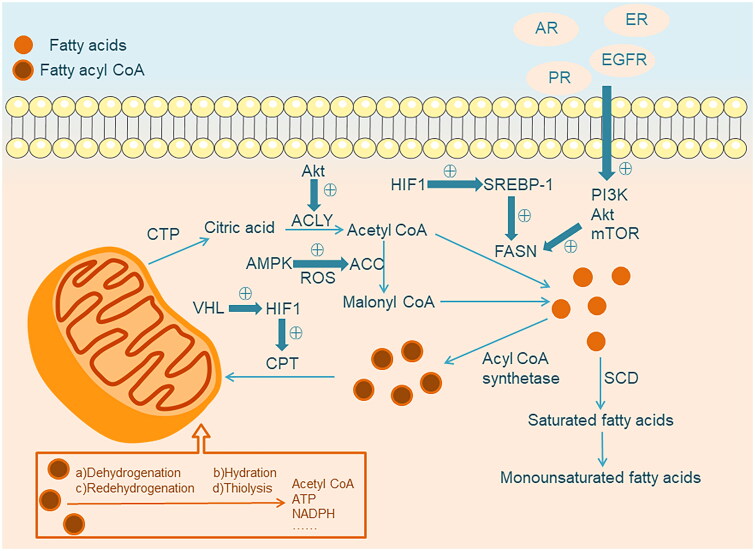
Remodeled fatty acid metabolism in tumor cells. The synthesis of FAs involves several key enzymes, including ACC, ACLY, and FASN. ACC carboxylates acetyl-CoA to form malonyl-CoA, while ACLY catalyzes the transfer of acetyl-CoA from the mitochondria to the cytoplasm for de novo FAs synthesis. The expression of FASN is regulated by EGFR, ER, AR, and PR. Downstream of these receptors, the PI3K-Akt-mTOR pathway is a candidate pathway. Dysregulated Akt can directly phosphorylate ACLY, leading to increased synthesis of acetyl-CoA. Hypoxia in the tumor microenvironment can also result in overexpression of FASN, possibly through Akt phosphorylation and activation of HIF1, which upregulates the transcriptional regulator SREBP-1. The presence of high levels of reactive oxygen species (ROS) also stimulates the upregulation of ACC by activating the AMPK signaling pathway. The β-oxidation of FAs occurs in three stages: activation, transfer, and oxidation. FAs are activated in the cytoplasm as acyl-CoA, transferred to the mitochondrial matrix via CPT, and oxidized to produce one molecule of acetyl-CoA and a new acyl-CoA, along with three times the energy generated by glucose oxidation. Thin blue arrows indicate biological processes and thick blue arrows indicate up-regulation.

**Figure 2.**
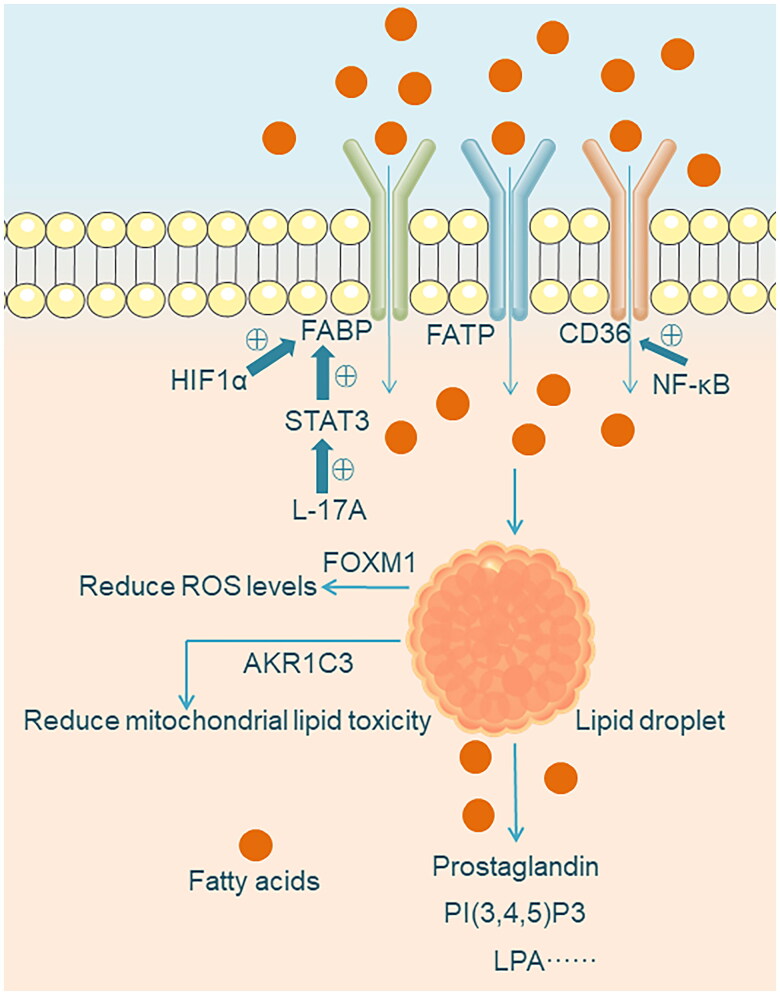
Extracellular uptake of FAs. The uptake of exogenous FAs by cells requires specific transport proteins such as CD36, FATP, and FABP. Under hypoxic conditions, the uptake of exogenous FAs mainly occurs through the overexpression of HIF-dependent lipid-binding proteins, with FABP4 being a transcriptional target of HIF1α. After uptake, exogenous FAs are primarily stored in LDs and can be released when needed for the synthesis of various oncogenic lipid signaling molecules such as PI(3,4,5)P3, prostaglandins, and LPA. LDs alleviates mitochondrial lipid toxicity and dysfunction in the form of AKR1C3 dependence. LDs accumulation reduces ROS levels and thus maintains endoplasmic reticulum homeostasis, which is associated with high expression of oncogenic transcription factor FOXM1.

The metabolism of FAs in tumor cells supports the need for rapid synthesis of nutrients such as ketone bodies, cholesterol and steroids, and provides a fuel source for the energy supply of tumor cells. The reprogramming of FA metabolism is a key aspect of tumor metabolism. In this section, we will focus on the detailed mechanisms of cancer cell’s reshaping of FA metabolism (Figures 1 and 2), with emphasis on three major steps: (1) de novo synthesis of FAs, (2) exogenous uptake of FAs, and (3) oxidation of FAs. In addition, ferroptosis is a newly defined programmed cell death process that is considered to be the link between abnormal metabolism and cell death, which we will discuss in this section.

### 2.1. Fatty acid synthesis

In most normal cells, de novo synthesis of FAs is not highly active, as the primary source of FAs is exogenous uptake. However, various tumor cells tend to shift from exogenous uptake to endogenous biosynthesis of FAs. As everyone knows, de novo synthesis of FAs involves three key enzymes, acetyl-CoA carboxylase (ACC), citrate transporter protein (CTP), and fatty acid synthase (FASN) [4]. In FAs synthesis, malonyl-CoA is required for FAs chain extension, and ACCs can catalyze the carboxylation of acetyl-CoA to produce malonyl-CoA, which provides three carbon compounds for FAs. ACC1 and ACC2 belong to ACC, where ACC1 is the first rate-limiting enzyme in the FAs synthesis pathway, and ACC2 produces malonyl-CoA to regulate the activity of CPT1 [5]. FASN is a multifunctional homodimeric enzyme protein. FASN monomer contains 6 kinds of catalytic activities. From the n terminal, there are β-ketoacyl synthetase (KS), acetyl/malonyl transacylase (AT/MT), β-hydroxylacyl dehydrase (DH), enoyl reductase (ER), β-ketoacylreductase (KR), acyl carrier protein (ACP) and thioesterase. FASN is the main enzyme required for the anabolic conversion of dietary carbohydrates into FAs. In the presence of NADPH, FAs synthases catalyze the conversion of acetyl-CoA and malonyl-CoA into long-chain saturated FAs [6]. CTP mediates the transport of citrate through the inner mitochondrial membrane and regulates the bidirectional transport of citrate between mitochondria and cytoplasm. Citric acid participates in the citrate - pyruvate cycle, transporting acetyl CoA into the cytosol, where acetyl CoA and oxaloacetic acid condense to form citric acid in mitochondria, and the latter is transported to the cytosol cleavage to interpret acetyl CoA for synthesis of lipoic acid and cholesterol. Citric acid can be used as an allosteric activator of acetylCoa carboxylase, and citric acid can cause it to undergo allosteric polymerization from inactive monomers to active polymers, thus promoting the synthesis of FAs [7,8].

In tumor cells, the upregulation of FAs synthesis is characterized by significantly increased expression and activity of various enzymes involved in lipid biosynthesis pathways. For example, transcription factors like sterol regulatory element-binding protein 1c (SREBP1c) and carbohydrate response element binding protein (ChREBP) synergistically regulate three key enzymes involved in FAs synthesis, including ACC, FASN, and CTP. Their coordinated regulation leads to enhanced FAs synthesis in tumor cells [9–12]. The increase in FASN expression and activity is observed in the early stages of tumorigenesis and is associated with cancer progression. Tumors that overexpress FASN exhibit a more invasive phenotype [5,13]. Existing studies have shown that FASN overexpression occurs in various human cancers, including prostate cancer, ovarian cancer, colon cancer, lung cancer, endometrial cancer, and gastric cancer, among others [14]. Transcriptional dysregulation of the FASN gene is one of the important mechanisms underlying its overexpression in tumor cells. The expression of FASN is regulated by various growth factors and signaling pathways, such as epidermal growth factor and its receptor (EGFR), estrogen and its receptor (ER), androgen and its receptor (AR), and progesterone and its receptor (PR). Downstream of these receptors, the PI3K-Akt-mTOR pathway is a candidate signaling pathway that mediates FASN expression [5,15]. In cancer, dysregulated Akt can directly phosphorylate and activate ATP-citrate lyase (ACLY), leading to increased synthesis of acetyl-CoA. Akt can also indirectly sustain the abundant production of FAs by activating nuclear factor-like 2 (Nrf2), a transcription factor, based on this, dysregulated PI3K/Akt pathway destroys cell homeostasis and is prone to carcinogenesis [16,17].

It is worth noting that the regulation of FASN expression by growth factors often involves mutual regulation. For example, human epidermal growth factor receptor 2 (HER2) induces FASN expression through downstream PI3K signaling. Conversely, the regulation of HER2 expression has been reported to be FASN-dependent. Inhibiting FASN leads to upregulation of PEA3 (a negative regulatory factor of HER2 gene transcription), resulting in decreased HER2 expression [18]. In addition, adverse conditions such as hypoxia and acidosis in the tumor microenvironment have also been reported to lead to the overexpression of FASN. This may be due to the phosphorylation of Akt and the activation of hypoxia-inducible factor 1 (HIF-1), which upregulates the transcriptional regulatory factor sterol regulatory element-binding protein-1 (SREBP-1) and further induce the expression of FASN [19,20].

In the initial step of FAs synthesis, citrate, an intermediate of the TCA cycle, is transported from the mitochondria to the cytoplasm by CTP for the release of acetyl-CoA. Similar to FASN, elevated levels of CTP are associated with tumor growth and invasion in various cancer cell lines. Recent studies have discovered that CTP plays a critical regulatory role in abnormal FA metabolism in human cancers. During metabolic stress, increased CTP enhances oxidative phosphorylation (OXPHOS) to protect cancer cells from energy stress-induced apoptosis. Inhibition of CTP activity can reverse lipid droplet formation and has inhibitory effects on tumor cell proliferation [21–23]. Furthermore, it is worth mentioning that under metabolic stress conditions such as hypoxia or lipid consumption, the presence of high levels of reactive oxygen species (ROS) stimulates the upregulation of acetyl-CoA synthetase 2 (ACSS2) in cancer cells. This leads to the generation of acetyl-CoA from acetate for FAs synthesis [24].

ACC1’s acetyl-CoA carboxylase alpha (ACACA) has been found to be highly expressed in prostate cancer tissue, and it has been found that in human castration-resistant PCa (CRPC) cell lines, knockout of ACACA can reduce FAs synthesis and down-regulate mitochondrial β-oxidation, resulting in reduced ATP production and increased reactive oxygen species, leading to apoptosis [25]. Unlike ACC1, ACC2 may play an inhibitory role in tumor invasion. Studies have found that ACC2 is significantly down-regulated in ovarian cancer, and this down-regulation promotes ovarian cancer proliferation and metastasis *in vitro* and *in vivo* by enhancing FAO, which can lead to tumor growth, metastasis, and worse prognosis [26]. Not only that, ACC can also hinder the impaired lipid metabolism of CD8+ T cells in the tumor microenvironment, and T cells in TME catabolize lipids through mitochondrial FAO to meet energy demands. However, Hunt et al. found that limiting ACC can change T cell metabolism, thereby maintaining energy under TME stress [27].

### 2.2. Fatty acid intake

Most normal cells, including those with relatively high proliferation rates, preferentially utilize FAs from dietary/exogenous lipids. Only a few normal tissues, such as adipocytes, hepatocytes, endometrial cells, and fetal pulmonary tissue cells, may have an active FAs synthesis process [28,29]. Therefore, in addition to de novo synthesis from nutrients such as glucose or glutamine, tumor cells can also directly uptake FAs from the surrounding microenvironment. This is particularly relevant in tumor microenvironments that lack sufficient nutrients. In this case, there may not be enough glucose to support the conversion of acetyl-CoA, which will lead to impaired de novo synthesis of FAs, and the process of converting saturated FAs to monounsaturated FAs may also be affected. This is when cancer cells need to increase their uptake of exogenous FAs [30].

Cellular uptake of exogenous FAs requires specific transport proteins to facilitate their transmembrane movement. Such carriers include CD36, also known as fatty acid translocase (FAT), fatty acid transport protein (FATP), and membrane fatty acid-binding protein (FABP). Studies have shown that their expression is significantly upregulated in cancers such as hepatocellular carcinoma and ovarian cancer, promoting the uptake of exogenous FAs. Under hypoxic conditions, the uptake of exogenous FAs primarily occurs through the overexpression of HIF-dependent lipid-binding proteins, with FABP4 being a transcriptional target of HIF1α [31,32]. High expression of CD36 is correlated with poor prognosis in various types of tumors, including breast cancer, ovarian cancer, gastric cancer, and prostate cancer. CD36 promotes the uptake and storage of FAs, as well as significant changes in lipid composition. The loss of CD36 is sufficient to slow down the growth of the aforementioned tumors [33–35]. In addition, CD36 can reduce cholesterol accumulation and ROS levels induced by adipocytes. CD36-mediated uptake of FAs is also closely associated with the induction of epithelial-mesenchymal transition (EMT), which is a key process in tumor tissue that confers both chemotherapy resistance and invasiveness [34,36]. Exogenously-derived FAs, after uptake, are primarily stored in lipid droplets (LDs). LDs serve as an inert cytoplasmic organelle, and the accumulation of LDs in cancer cells is not only essential for maintaining lipid homeostasis but also provides ATP and NADPH under metabolic stress conditions [37,38]. FAs stored in LDs are released when needed. As a precursor for lipid synthesis, these released FAs can be utilized for the biosynthesis of various oncogenic lipid signaling molecules. For example, PI(3,4,5)P3 is a lipid product formed through the involvement of phosphoinositide 3-kinase (PI3K), which activates the Akt signaling pathway to stimulate cancer cell proliferation and survival [39]. Similar examples include lysophosphatidic acid (LPA), which signals through the G protein-coupled receptor family to promote adhesion, migration, and invasion of ovarian cancer cells [40]. Prostaglandins are a class of lipid messengers formed by the catalysis of cyclooxygenase. They can promote cancer cell migration and tumor-host interactions. The synthesis of prostaglandins is also associated with the release of FAs from LDs [41].

### 2.3. Fatty acid oxidation

Most tumor cells exhibit a higher glucose uptake rate to support their increased proliferation and biosynthetic demands. However, in prostate cancer cell lines and breast cancer cell lines, there is an increased dependence on FAs β-oxidation, with FAs serving as the primary energy source. As a result, these cells exhibit lower glucose consumption rates, and there is an upregulation of enzymes involved in β-oxidation [42,43]. Therefore, the reprogramming of FAO is another safeguard for the survival of tumor cells.

In normal tissues, the liver and muscles are the most active sites for FAO, with the primary form being beta-oxidation. Beta-oxidation breaks down FAs into CO_2_ and H_2_O, releasing a significant amount of energy. Enhanced FAO has been observed in various types of cancer. For example, in glioblastoma, high expression of the neural stem cell growth factor (ACBP) has been observed. ACBP binds to acyl-CoA, promoting FAO to meet the high demand of tumor cells for ATP and synthetic metabolism [44]. The beta-oxidation of FAs consists of three stages: activation, transfer, and oxidation. In the cytoplasm, FAs are activated in the form of fatty acyl-CoA. They are then transferred to the mitochondrial matrix *via* carnitine palmitoyltransferase (CPT), where they undergo oxidation to generate one molecule of acetyl-CoA and a new fatty acyl-CoA, along with about twice the amount of energy produced by glucose oxidation. CPT is a key regulator of FAO and serves as an important regulatory factor in tumor cells, playing a role in proliferation and apoptosis along with other critical pathways such as Src regulation and VEGF signaling [45,46].

Research shows that CPT can activate FAO, increase ATP and NADPH production, and protect tumor cells from the effects of environmental stress such as glucose deprivation and hypoxia. CPT is closely associated with the progression of various cancers, including breast cancer, gastric cancer, prostate cancer, lung cancer, ovarian cancer, liver cancer, multiple myeloma, and glioblastoma [47]. Hypoxic environments are the main obstacle to tumor cell growth. They induce HIF-1-dependent lipid droplet accumulation and slow down FAO in tumor cells. HIF-1α inhibits two enzymes involved in FAO, including medium-chain acyl-CoA dehydrogenase (MCAD) and long-chain acyl-CoA dehydrogenase (LCAD). The loss of LCAD further promotes cancer development [48,49]. In breast cancer, it has been observed that hypoxic environments inhibit FAO. However, antioxidants such as L-carnitine can protect cancer cells from ROS-induced cytotoxicity by enhancing the activity of CPT [50,51]. Significantly upregulated CPT promotes tumor cell adaptation to metabolic stress and resistance to mTOR complex 1 (mTORC1) inhibitors. Under conditions of *in vitro* environmental stress, it increases tumor cell survival rates [52,53]. In addition, CPT has been shown to inhibit tumor cell apoptosis by promoting tumor neovascularization, clearing toxic lipid metabolites, and activating the Bcl-2 protein. These actions further promote tumor growth [47].

It is worth noting that although most cancers exhibit rewiring of FA metabolism, different cancer types seem to have their tendencies. For example, the receptor-positive breast cancer (RPBC) and triple-negative breast cancer (TNBC) subtypes show significant differences in FAs uptake, storage, and oxidation. RPBCs tend to overexpress genes related to FAs synthesis, lipid mobilization, and β-oxidation, such as PGC1α, CPT1B, and CDCP1. On the other hand, TNBCs tend to overexpress genes related to exogenous lipid uptake [54]. We will discuss the differences in FA metabolism rewiring among various cancers and subtypes in subsequent sections.

### 2.4. Desaturation of fatty acids and elongation of fatty acid chains

FAs desaturation and chain elongation also play a vital role in the genesis and development of tumor cells. A decrease in the ratio of polyunsaturated FAs to saturated FAs (P/S) in cell membranes may lead to decreased membrane fluidity and impaired function. Over-saturated palmitic acid binds to phospholipids and TG of cell membranes to increase the content of saturated lipids, and the accumulation of saturated FAs and neutral lipids in non-adipose tissues can rapidly stimulate cell apoptosis [55]. Tumors respond to this lipid toxicity by altering the activity of enzymes associated with lipid metabolism. Stearoyl-coa desaturase 1 (SCD1) is a key enzyme in FAs desaturation, and earlier studies have found that high expression and activity of SCD1 exist in the pathogenesis of many tumors [56]. Therefore, FAs desaturation reprogramming may promote tumor survival and progression. FAs desaturation hassss been found in a variety of cancers, which not only promotes the development of tumors, but may even have carcinogenic effects. At present, researchers have used this mechanism to selectively inhibit related pathways to inhibit tumor growth. Won et al. found that a new eicosenoic acid is produced by changing the desaturation of FAs through SCD, which promotes excessive proliferation and survival of undesirable cells. SCD inhibitors can selectively target underdeveloped cells of gastric organoids *in vivo*, which may inhibit the occurrence of gastric cancer [57]. SREBP1 is overexpressed in clear cell renal cell carcinoma (ccRCC), which can drive lipid desaturation and promote the activation of NF-κB, thereby promoting tumor cell growth. Inhibition of NF-κB can block the growth-promoting function of SREBP1 in ccRCC [58]. Another study using paper omics analysis of ccRCC tissue found significant accumulation of PUFAs and observed increased expression of SCD1, and subsequent treatment with a small molecule SCD1 inhibitor (A939572) was found to delay the rate of renal cancer cell proliferation [59]. In breast cancer, SCD1 inhibits tumor cell apoptosis by inducing endoplasmic reticulum stress and leading to apoptosis through activation of the unfolded protein response (UPR) sensor. The addition of products of exogenous SCD1 insulates epithelial ovarian cancer (EOC) cells from this apoptosis, underscoring the importance of lipid desaturation for cancer cell survival [60].

FAs chain elongation can be produced by unsaturated FAs catalyzed by related enzymes [61]. FAs extension enzyme expression is increased in many cancers, such as pancreatic cancer, prostate cancer, kidney cancer, rectal cancer and so on. This suggests that FAs extension may play a role in cancer progression. By comparing Elongation of very long chain fatty acid (ELOVL) in normal colon mucosa and colorectal cancer samples, Mika et al. found that ELOVL1 and ELOVL6 were more strongly expressed in the latter, and the level of very long-chain FAs was significantly increased [62]. In pancreatic cancer stem cells (PCSCs), the expression of the three-functional enzyme subunit α is upregulated, which is mainly involved in fatty acid elongation and unsaturated FAs biosynthesis, lipidomics reveals an increase in long and very long chain unsaturated FAs, and ELOVL5 is predicted to be a key gene [63]. In response to these phenomena, current studies have focused on targeting fatty acid lengthening enzymes to reverse tumor proliferation. One study found that ELOVL5 deletion in prostate cancer significantly altered mitochondrial morphology and function, leading to overproduction of reactive oxygen species, which inhibited prostate cancer cell proliferation and metastasis, and conversely, supplementing the direct product of ELOVL5 extension could reverse this [64]. Similar findings were also found in kidney cancer. Nitta et al. inhibited the formation of lipid droplet through CRISPR/Cas9-mediated ELOVL5 knockdown, induced apoptosis through endoplasmic reticulum stress, and then inhibited the proliferation and growth of tumor cells [65].

### 2.5. Fatty acids metabolic reprogramming and ferroptosis in tumor cells

**Figure 3.**
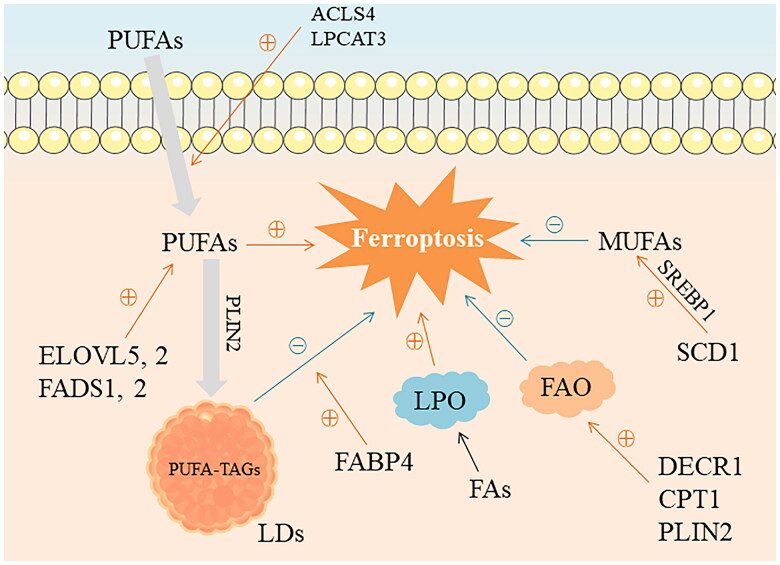
Metabolic remodeling of FAs mediates tumor-related ferroptosis. Free PUFAs can be bound to cell membranes by various enzymes, including long-chain ACLS4 and Lysophosphatidylcholine acyltransferase 3 (LPCAT3). The content of PUFAs in tumor cells is the key factor to induce ferroptosis. Enzymes involved in PUFAs synthesis and metabolism include ELOVL5, ELOVL2, FADS1, and FADS2. Compared with PUFAs, MUFAs has the effect of protecting cells from ferroptosis. Stearoyl-CoA desaturase 1 (SCD1) is primarily used for the production of MUFAs and depends on the activation of terol regulatory element-binding protein 1 (SREBP1). FAO inhibits ferroptosis in cancer cells in a glutathione-independent manner, and enzymes involved in FAO, including 2,4-dienoyl-CoA reductase 1 (DECR1), carnitine palmitoyltransferase 1 (CPT1), PLIN2, and others, play a key role in this. LDs protects cells from oxidative stress by regulating the transport of PUFAs, and PUFAs can accumulate in LDs in the form of PUFA-TAGs, in which PLIN2, a protein associated with lipid differentiation, wraps around LDs along with phospholipids and is involved in assisting the storage of neutral lipids in LDs. FABP4 enhances LDs function in cancer cells and protects cancer cells from oxidative stress-induced iron death. Black arrows indicate participation in biological processes, orange arrows indicate up-regulation, and blue arrows indicate down-regulation.

Ferroptosis is an iron-dependent programmed cell death, which is different from apoptosis, necrotic apoptosis, and pyroptosis in morphology and mechanism. The accumulation of lipid peroxidation (LPO) and peroxides is a sign of ferroptosis, which can directly damage the cell membrane and lead to cell dysfunction, thus inducing ferroptosis. Thus, metabolic remodeling of FAs plays a key role in inducing ferroptosis in tumor cells [66] (Figure 3).

Free PUFAs can be incorporated into cell membranes by various enzymes such as long-chain acyl-CoA synthetase 4 (ACLS4) and Lysophosphatidylcholine acyltransferase 3 (LPCAT3), while phospholipids containing PUFAs are the main substrates for LPO in ferroptosis, including PE and phosphatidylcholine (PC). The content of PUFAs in tumor cells is one of the key factors in ferroptosis [66,67]. The study found that PUFAs selectively induced iron death of cancer cells in an acidic environment, thereby delaying tumor growth. In addition, in pancreatic cancer tissue, PUFAs increased LPO of pancreatic cancer cells, enhanced the phosphorylation levels of receptor-interacting protein kinase 3 (RIP3) and mixed-lineage kins-like domain (MLKL) and induced iron death of pancreatic cancer cells. ferrostatin 1, an iron death inhibitor, reverses this effect [68,69]. Enzymes involved in PUFAs synthesis and metabolism, such as ELOVL5, ELOVL2, fatty acid desaturase 1 (FADS1), and FADS2, play an important role in ferroptosis. ELOVL5 is primarily responsible for extending the 18-carbon FAs to 20 carbons, while FADS1, as a key rate-limiting enzyme in PUFA metabolism, can catalyze dihomo-gamma-linolenic acid to arachidonic acid. In gastric cancer, enteric gastric cancer cells express very low levels of ELOVL5 and FADS1 due to hypermethylation of the promoter region and are therefore resistant to ferroptosis, and their sensitivity to ferroptosis can be restored after PUFAs supplementation, while mesenchymal GC cells are sensitive to ferroptosis due to high levels of ELOVL5 and FADS1 [70,71]. Compared with PUFAs, MUFAs can protect cells from ferroptosis. For example, lipidomics showed that oleic acid (OA) can reduce the level of ferroptosis-related phospholipids and effectively inhibit the iron-dependent oxidative cell death process. This protective effect is also associated with inhibiting the accumulation of lipid-reactive oxygen species on the plasma membrane [72]. SCD1 is mainly used for the production of MUFAs and depends on the activation of SREBP1. Inhibition of SCD1 or SREBP1 will block MUFA synthesis, increase the PUFA/MUFA ratio, and make tumor cells sensitive to ferroptosis. Upregulating the expression of SCD1 or SREBP1 also restored their resistance to ferroptosis [73]. Similarly, a diet rich in long-chain PUFAs in mice can significantly delay tumor growth by increasing the PUFA/MUFA ratio and inducing ferroptosis in tumor cells under acidosis conditions. In contrast, MUFAs rich in lipoproteins in the diet remodeled cell membranes, reducing the PUFA/MUFA ratio and protecting melanoma cells from ferroptosis [68,74].

In addition, CD36 mediates the uptake of FAs by tumor-infiltrating CD8^+^ T cells, inducing LPO and ferroptosis, resulting in reduced tumor-toxic cytokine production and impaired anti-tumor immunity. Blocking CD36 or inhibiting the ferroptosis process of CD8^+^ T cells can effectively restore their antitumor activity [75]. In FAO’s case, another essence of ferroptosis is glutathione (GSH) depletion, so FAO inhibits ferroptosis in cancer cells in a GSH-independent manner. Thus, inhibition of FAO, such as knocking out 2,4-dienoyl-CoA reductase 1, a β-oxidizing coenzyme, makes prostate cancer cells sensitive to ferroptosis, while upregulation of FAO, such as upregulation of CPT1 activity, improves ferroptosis-induced placental damage in mice by correcting the FAO/peroxide imbalance [76–78]. Unused FAs in cells can be stored in LDs in the form of triacylglycerides (TAGs), which may have multiple functions in terms of ferroptosis, including regulating phospholipid synthesis pathways, modifying membrane lipid homeostasis, and preventing lipid toxicity. LDs protect cells from oxidative stress by regulating the transport of PUFAs. Specifically, PUFAs can accumulate in LDs in the form of PUFA-TAGs instead of forming phospholipids in membrane lipids, which prevents PUFA oxidation from occurring in the cell membrane and makes the cells less susceptible to ferroptosis [79–81]. For example, Perilipin2 (PLIN2) is a lipid differentiation-associated protein that, together with phospholipids, surrounds the lipid droplet and is involved in assisting the storage of neutral lipids in the lipid droplet. In gastric cancer, PLIN2 alleviates the metabolic abnormality of FAs, inhibits the ferroptosis process in gastric cancer cells, and enhances proliferation [82].

In addition, fatty acid desaturation and chain elongation were closely related to iron death of tumor cells. FAs desaturation can protect tumor cells from oxidative stress-induced iron death. The coordinated activation of transcription factor CCAAT-enhancer binding protein α (C/EBP-α) and Fms-like tyrosine kinase 3 (FLT3) is known to promote the biosynthesis and desaturation of FAs. FLT3 or C/EPPα inactivation reduces monounsaturated FAs incorporation into membrane phospholipids through SCD downregulation, enhances its sensitivity to lipid REDOX stress, and triggers lipid oxidative stress by combining FLT3 and glutathione peroxidase 4 inhibition. Thereby enhancing iron death in FLT3 mutated acute myeloid leukemia (AML) cells [83]. Another study found that SCD1 causes FAs desaturation, while fatty acid-binding protein-4 (FABP4) derived from TEM enhances LDs in cancer cells, which together protect cancer cells from oxidative stress-induced iron death, and fatty acid desaturation triggers the tumor’s intrinsic antioxidant and anti-iron death resources. Helps tumors survive and regenerate [84]. As for the effect of fatty acid chain extension on iron death of tumor cells, Tian et al. studied the effect of overexpression of ELOVL6 on CRC cells. In this study, apatinib was used to treat CRC HCT116 cells and found that it increased the content of intracellular iron reactive oxygen species. Overexpression of ELOVL6 reversed the effects of HCT116 cell viability and iron death [84].

Overall, FAs components of tumor cells play pro-oxidant/anti-oxidant roles during their ferroptosis, which may be a unique mechanism by which FAs metabolic reprogramming influences cancer progression.

## 3. Changes in signal related to fatty acid metabolism in different cancers

**Figure 4.**
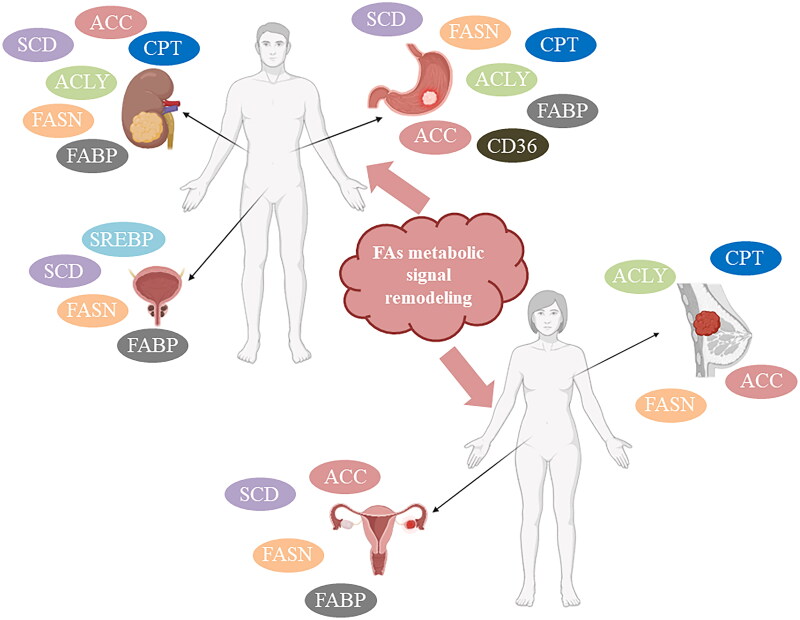
Dysregulated fatty acid metabolism signaling in gastric cancer, BC, ovarian cancer, prostate cancer, and renal cancer. The dysregulation of fatty acid metabolism (FAs) may be one of the contributing factors to the development and progression of various cancers. We have summarized the aberrant FA metabolism signaling in 5 types of human cancers, such as the dysregulated expression of SCD, FASN, CPT, ACLY, FABP, ACC, and CD36 in gastric cancer. We will provide further details on these mechanisms in Table 1.

It is well known that the main enzymes involved in FAs synthesis are ACLY, ACSs, ACC, FASN, and SCD1, with SREBP1 being an upstream molecule. FA uptake is primarily mediated by CD36 and FABPs, while FAO is mediated by CPT1. The rewiring of FA metabolism in various human cancers is reflected in alterations at each step (Figure 4). Here, we will discuss the reshaping of FA metabolism at various steps in several typical cancers and summarize the published research on the consequences of abnormal FA metabolism in different cancers (breast cancer, prostate cancer, renal cell cancer, ovarian cancer, stomach cancer, liver cancer, lung cancer, and colorectal cancer) (Table 1).

**Table 1.** The mechanism and experimental evidence of FAs metabolic reprogramming affecting the occurrence and development of different types of cancer.

Cancer	Factor	Level	Model	Mechanism	References
Ovarian cancer	FASN	up regulation	Human OC cell line Skov-3	Promote cell growth and vitality, inhibit cell apoptosis, inhibit tumor infiltration DC, and weaken anti-tumor immunity.	[1,2]
	SCD1	up regulation	Human OC cell line TOV-112D	Promote the expression of stem cell transcription factors, promote the growth of OC stem cells, inhibit lipid oxidation, and inhibit cell iron death.	[3,4]
	FABP4	up regulation	Human OC cell line Ovcar-3	Activate β-catenin pathway, down-regulate PTEN, and increase cancer cell adhesion.	[5]
	SIK2	up regulation	C57BL/6 mice	Promote ACC phosphorylation and activation of PI3K/AKT pathway, regulate fatty acid oxidation and greater omental metastasis of OC.	[6]
	CPT1A	up regulation	Human OC cell line Ovcar-3	Increase the level of β oxidation and ATP of fatty acids, promote cell proliferation.	[7]
	ACLY	up regulation	Human OC cell line A2780	Accelerate the cell cycle process and promote the proliferation of cancer cells.	[8]
Prostatic cancer	FASN	up regulation	Human PCa cell line LNCaP	Promote the formation of pseudopods, activate the production of arachidonic acid and androgens, and promote tumor migration, adhesion and invasion.	[9]
	SREBPs	up regulation	Normal prostate cell lines (RWPE-1), PCa cell lines (LNCaP)	Promote clonal generation, invasion and migration; Increase the percentage of CD44+/CD24- subpopulation to promote stem cell production; Targeting c-Myc induces stem cell formation; Promote EMT and increase invasiveness.	[10]
	SCD1	up regulation	Human PCa cell lines RWPE-1	Promote cell proliferation through AR dependent pathway; Affect lipid metabolism, promote the proliferation of cancer cells and fat synthesis.	[11,12]
	ELOVL5	up regulation	Human PCa cell lines LNCaP	Promote fatty acyl chain extension of cancer cells, reduce mitochondrial oxidative stress, maintain appropriate ROS levels, and promote tumor occurrence and metastasis.	[13]
	FABP5	up regulation	Human PCa cell lines LNCaP	Transport lipid ligands to nuclear receptors (PPARγ), promoting metastasis.	[14]
Renal cell carcinoma	FASN	up regulation	Human RCC cell lines A498 and 786 O, clinical tumor samples	Promote invasive cell proliferation, migration, apoptosis and lipid droplet formation.	[15]
	ACLY	up regulation	Human RCC cell line GRC-1, clinical tumor samples and paracancer tissue	Promote mitochondrial lipogenesis and inhibit cell apoptosis; Leads to lipid deposition and promotes cell proliferation.	[16,17]
	SCD1	up regulation	Human RCC cell lines RWV366T	Promote cell proliferation, destroy endoplasmic reticulum homeostasis, and inhibit cell apoptosis.	[18]
	FABP5	up regulation	Human RCC cell lines Caki-1	Up-regulation of p-AKT and activation of PI3K/AKT signaling pathway promote cell proliferation and lead to malignant progression of tumors.	[19]
	CPT1A	down regulation	Human RCC cell lines 786-O	Reduce fatty acid transport to mitochondria, promote lipid droplet formation, and limit tumor growth.	[20]
Gastric carcinoma	SREBP1c	up regulation	Human GC cell lines AGS	Promote the expression of fatty acid synthesis-related genes, reduce palmitic acid concentration, and promote cell proliferation, invasion and migration.	[21]
	ACSS2	down regulation	Clinical tumor samples	Independent factor of poor prognosis of gastric cancer.	[22]
	ACSS3	up regulation	Human GC cell lines SNU1	Promote the expression of transcription factors related to starvation signals, and promote cell growth and invasion.	[23]
	FASN	up regulation	Bioinformatics analyses	The regulation of macrophage polarization leads to tumor immune infiltration and promotes tumor immune escape.	[24]
	ACC	up regulation	Multi-omics GC datasets	Reduce CD8+ T cell enrichment and inhibit anti-tumor immunity.	[25]
	SCD1	up regulation	Human GC cell lines SGC-7901	Up-regulate the expression levels of Yap and β-catenin, promote proliferation; The expression of SLC7A11 and GPX4 was enhanced, and iron death was inhibited.	[26]
	CPT1A	up regulation	The gastric mucosal epithelial cell line GES-1 and human GC cell lines	Increase NADP+/NADPH ratio and activate FAO in GC cells; Promote EMT.	[27]
Breast cancer	ACLY	up regulation	Human BC cell lines MCF7, clinical tumor samples	Interaction with Cyclin E leads to the formation of lipid droplets in cells, promoting cell transformation, migration and invasion.	[28]
	ACC	up regulation	Female 4-week-old nude mice, human BC cell lines MCF7	Promote EMT, enhance cell viability and migration ability, reduce the expression of Bax and inhibit cell apoptosis.	[29]
	FASN	up regulation	Human BC cell lines HBL100	It activates the HER1/HER2 tyrosine kinase receptor and promotes cell proliferation, invasion and migration.	[30,31]
	CPT1A	up regulation	Human BC cell lines MCF7	Promote the formation of mammary glands; Through the amplification of chromosome 11q13-14, it promotes the occurrence and progression of tumor.	[32]
	SCD1	up regulation	Human BC cell lines MCF7	Leads to the formation of oleic acid, promotes cell survival and migration.	[33]
Liver cancer	FASN	up regulation	Human normal liver cell line 7702 and hepatoma cell lines HepG2, clinical tumor samples	Promote cell proliferation, invasion and migration.	[34]
	ACLY	up regulation	Human hepatoma cell lines Huh7, clinical tumor samples	Increase the stability and protein level of β-catenin, regulate stem-ness of Liver tumor initiating cells through Wnt/ β-catenin signal, and promote cell migration.	[35]
	SCD1	up regulation	Human hepatoma cell lines HepG2	Activate phosphatidylinositol 3 kinase/c-Jun N-terminal kinases to promote the progression of liver cancer and chemotherapy resistance.	[36]
	SREBP1	up regulation	Human immortal liver cell line LO2 and hepatoma cell lines HepG2, clinical tumor samples	Promote FASN, ACC, and HMGCR gene transcription; promote cell proliferation, migration and invasion, resulting in chemotherapy resistance.	[37]
Lung cancer	FASN	up regulation	Human NSCLC cell lines A549 and NCI-H1299	Activate AKT/ERK pathway, participate in glucose metabolism, and change the malignant phenotype of lung cancer cells.	[38]
	SREBP1	up regulation	Human NSCLC cell lines H441and A549	Maintains stemness of cells and enhances the ability of cells to form spheres, leading resistant cells to develop CSC-like phenotypes.	[39]
	SCD1	up regulation	Human lung adenocarcinoma cells H460	Change the levels of cyclin D1 and CDK6 to control the cell cycle process; Inhibition of toxic or fat apoptosis mediated by saturated fatty acids; Stimulate the biosynthesis of cis-monounsaturated fatty acids and promote cell proliferation.	[40]
	ACLY	up regulation	Human lung adenocarcinoma cell lines A549	Increase the conversion rate of glucose to lipids and promote cell proliferation.	[41]
Colorectal cancer	FASN	up regulation	Human CRC cell lines SW480, normal colon epithelial cells NCM460, clinical tumor samples	Increased ATP production leads to AMPK inhibition and mTOR activation, promoting the proliferation and metastasis of colorectal cancer cells.	[42]
	SREBP1	up regulation	Human CRC cell lines HCT116 and SW480, clinical tumor samples	The expression level of caspase-7 protein was down-regulated, apoptosis was inhibited, and chemotherapy resistance to gemcitabine was induced.	[43]
	SCD1	up regulation	Human CRC cell lines HCT116	Regulate EMT, promote cell migration and invasion; Under the condition of high glucose, PTEN/Akt signal is inhibited by MUFA, EMT is regulated, and cell migration and invasion are promoted.	[44]
	FABP6	up regulation	Bioinformatics analyses	It is associated with insulin-like growth factor signaling and is involved in cell proliferation.	[45]

### 3.1. Ovarian cancer

An increasing number of studies suggest that lipid metabolism dysregulation is a key factor in the progression of ovarian cancer (OC), and plasma FAs levels and composition are potential biomarkers for OC. Firstly, FASN is upregulated in OC cell lines and OC tissues, promoting FAs synthesis, cell growth, and cell viability. This is closely associated with tumor grade, poor prognosis, and drug resistance [85–88]. There are also reports that FASN impedes the anti-tumor immune process in OC. Specifically, overexpression of FASN in mouse ovarian epithelial cell lines leads to abnormal accumulation of unsaturated fatty acids, SFAs, and TG, resulting in dysfunctional tumor-infiltrating dendritic cells [89].

Additionally, SCD1 regulates the composition of FAs in tumors, and its expression is upregulated in OC stem cells. The desaturation of lipids is closely associated with the metabolic activity of OC stem cells. NF-kB is an upstream target that directly regulates the transcription of SCD1. Treatment with SCD1 inhibitors can inhibit the growth of OC stem cells. Furthermore, upregulation of SCD1 can protect OC cells from iron-induced cell death, suggesting that targeting SCD1 may be a potential therapeutic strategy for OC [73,90,91]. Yang et al. found that the combination of SCD1 inhibitors with cisplatin can inhibit peritoneal dissemination of OC cells and restore their sensitivity to iron-induced cell death [92].

In terms of FA oxidation and intake, FABP4 regulates the FA metabolism of OC cells by disrupting tumor-infiltrating dendritic cells, thereby interfering with the anti-tumor immune environment, which often leads to widespread metastasis and poor prognosis of OC [32]. Salt-inducible kinase 2 (SIK2), a member of the AMPK family, is upregulated in adipose tissue and regulates FAO mediated by PI3K and ACC1, thereby promoting abdominal metastasis of OC [93].

In addition, active inflammatory cytokines in OC are closely associated with FA metabolism. IL-17A promotes the growth and metastasis of OC by regulating tumor cell uptake of FAs. IL-17A has also been found to induce FABP4 expression and promote FAs uptake by activating phosphorylation of STAT3, thereby facilitating the metastasis of OC cells. Moreover, IL-1β and TGF-β1 stimulate the release of saturated FAs from adipocytes, leading to the production of pro-inflammatory mediators and conferring chemoresistance to tumor cells [94,95]. IL-6, IL-8, monocyte chemoattractant protein-1 (MCP-1), and tissue inhibitor of metalloproteinase-1 (TIMP-1) promote tumor cell growth by activating FAs synthesis, possibly through the involvement of the PI3K/AKT pathway and peroxisome proliferator-activated receptor (PPAR) signaling. PPARs belong to the nuclear receptor family, and their activation is a hallmark of cancer progression [90,96].

### 3.2. Prostatic cancer

Extensive alterations in lipid metabolism are considered hallmark features of prostate cancer (PCa). PCa tissues exhibit high concentrations of FAs due to dual upregulation of uptake and synthesis. Increased FAs uptake mediated by CD36 is associated with PCa invasiveness, and silencing CD36 in PCa cells can reduce cell proliferation and decrease cancer severity [35]. Additionally, the upregulation of FAs synthesis in PCa is accompanied by overexpression of FASN and predicts higher Gleason scores and pathological stages [97].

One of the unique aspects of PCa is the involvement of the androgen receptor (AR). AR plays a crucial role in PCa growth and progression by regulating epithelial differentiation and downstream gene expression. Dysregulated AR signaling is closely associated with PCa growth and progression. As a ligand-dependent nuclear transcription factor, AR can activate sterol regulatory element-binding proteins (SREBPs), which play a central role in FASN expression. This may be one of the reasons for the high malignancy of castration-resistant prostate cancer (CRPC) [97–99]. Furthermore, androgen receptor splice variant 7 (AR-V7) is upregulated in PCa with bone metastasis, consistent with variations in FASN expression. The FASN inhibitor IPI-9119 can inhibit the growth of AR-V7-driven CRPC and enhance sensitivity to enzalutamide in a xenograft model [98]. SCD1 promotes PCa cell proliferation through an AR-dependent pathway, and the SCD1 inhibitor BZ36 can inhibit this phenomenon through the PI3K/AKT-dependent pathway [100,101]. ELOVL5, a member of the elongase protein family, is a key enzyme involved in the production of PUFAs and is a critical factor in PCa’s tumorigenic metabolism. Its activity is regulated by androgens and is crucial for PCa metastasis and growth [64]. Fatty acid binding protein 5 (FABP5) is involved in the uptake, transport, and metabolism of FAs in the cytoplasm. Its expression is upregulated in PCa and promotes PCa metastasis, mainly through PPARs and estrogen receptor α (ERα)-mediated regulation of EMT [102–104]. α-methyl acyl CoA racemase (AMACR) is also an important enzyme involved in FAO and is specifically overexpressed in colorectal cancer, liver cancer, and prostate cancer. In particular, AMACR is significantly upregulated in prostate cancer and has been proven to be an effective biomarker for early diagnosis in prostate cancer patients [54,105].

### 3.3. Renal cell carcinoma

Renal cell carcinoma (RCC) is characterized by high metabolic reprogramming, and dysregulation of FA metabolism is one of the most significant changes in RCC. Increased endogenous FAs synthesis or exogenous FAs uptake is necessary for the survival and proliferation of RCC tumor cells, which may be associated with their invasiveness [106]. In RCC, inactivation of the AMPK-GATA3-ECHS1 pathway induces FAs synthesis and accumulation. Specifically, ECHS1 downregulation inhibits the expression of AMPK-promoted ECHS3 transcriptional activator GATA1, leading to the accumulation of FAs and branched-chain amino acids (BCAAs), thus promoting RCC cell proliferation [107].

In addition, lipid omics research has shown an increased utilization of FAs in RCC [108], which may be attributed to a series of dysregulations in FA metabolism: (a) Overexpression of FASN in RCC, similar to other malignancies, is associated with aggressiveness and poor prognosis [109]; (b) Increased expression of ACLY in RCC tumor tissue compared to adjacent normal tissue, and its artificial downregulation can inhibit RCC cell proliferation and induce apoptosis [110]; (c) Elevated levels of ACC protein are correlated with poorer clinical outcomes in RCC patients, and chemical inhibition of ACC selectively induces growth arrest and cytotoxicity in RCC cells [111,112]; (d) SCD expression is also higher in malignant renal cells of RCC patients compared to adjacent normal tissue, and patients with higher SCD expression have lower overall survival rates [113,114]; (e) Upregulation of FABP-5 in RCC promotes cell proliferation and is associated with poorer overall survival rates in patients, particularly through the PI3K/AKT pathway [115].

Furthermore, the hypoxia-inducible factor-controlled FA metabolism plays a crucial role in the development of RCC. Specifically, alterations in the von Hippel-Lindau (VHL) tumor suppressor stabilize HIF, which is the most common molecular characteristic in RCC. The direct target gene of HIF, CPT1A, is inhibited by both HIF1 and HIF2, leading to a decrease in the transport of FAs into the mitochondria and forcing the storage of FAs in LDs. Functionally, the inhibition of CPT1A is essential for tumor formation, and adverse patient outcomes are associated with lower expression of CPT1A in tumors. Therefore, restoring CPT1A levels in RCC cell lines can reduce the number of LDs and inhibit tumor growth [116].

Moreover, lipids are involved in the regulation of the metabolic process of cancer stem cells (CSC) in renal cancer, and their metabolism can be transformed from aerobic glycolysis to OXPHOS under hypoxia conditions, which is associated with more LDs and CD133+ cell expression in CSCs, thus enhancing the clonability of tumor cells [117]. Fatty acid metabolism can also affect the tumor microenvironment of renal cell carcinoma, thereby altering tumor progression and cancer treatment-related progression. The gene CPT1B, which is related to fatty acid metabolism, was positively correlated with T cells, CD8^+^T cells, cytotoxic lymphocytes and NK cells in the RCC tumor microenvironment, and negatively correlated with myeloid dendritic cells, fibroblasts and endothelial cells, and knockdown of CPT1B significantly inhibited the proliferation of RCC cells [118]. Studies have shown that somatic mutations in PBRM1 and KDM5C are associated with FAO gene expression, and these changes promote RCC angiogenesis [119]. A G-protein-coupled receptor, free fatty acid receptor-4 (FFA4), which is intrinsically activated by medium-long chain free FAs, is upregulated in RCC, and it has been found that FFA4 and selective agonists are positively regulated through PI3K/AKT/NF-κB signaling of COX-2 and MMP-9. EGFR is trans-activated, which intensifies tumor cell migration and invasion. Changes in the tumor microenvironment affect the biology of the disease and may affect response to systemic therapy [120]. Lin et al. show that upregulated FAO promotes malignant features of ccRCC cells through the JAK1/STAT3 signaling pathway and M2-like macrophage polarization. This suggests that targeting fatty acid metabolism affects the therapeutic efficacy of PD-1/PD-L1 in TME and related signaling pathways [121].

### 3.4. Gastric carcinoma

Gastric cancer (GC) is an extremely invasive tumor, and FAs serve as a vital substrate for its rapid proliferation and biosynthesis. Therefore, dysregulation of essential molecules involved in FA metabolism is closely associated with GC progression [122]. SREBP1 is upregulated in GC tissues, likely stimulated by HIF-1α, and the upregulated SREBP1 affects the expression of ACC, FASN, and SCD1. Inhibiting SREBP1 can significantly suppress the proliferation and migration of GC cells [123–125]. For example, Apatinib effectively treats multi-drug resistant GC cells by mediating the binding of SREBP1A to the GPX4 promoter region, thereby regulating GPX4 transcription [126].

The ACLY gene is located on chromosome 17q and exhibits significant amplification in GC tissues, and it is associated with advanced-stage, Helicobacter pylori positivity, lymph node metastasis, and poor prognosis [127–129]. ACLY activity can be inhibited by sodium citrate, miR-133b, and lncRNA FLJ22763, thereby suppressing the growth and invasion of GC cells [130,131]. The short-chain acyl-CoA synthetase (ACSS) family, which consists of ACSS1, ACSS2, and ACSS3, is responsible for converting acetate to acetyl-CoA, and all three members exert target effects in GC. The methylation level of the ACSS1 promoter in primary EBV(+) GC is significantly higher than that in EBV(-) GC. Deficiency in ACSS2 expression is an independent prognostic factor for the survival period of GC patients, indicating a poor prognosis. ACSS3 serves as a prognostic marker for GC and its knockout may reduce colony formation in conventional culture and increase tumor cell death rate [132–134].

As a rate-limiting enzyme in fatty acid synthesis, ACC is significantly negatively correlated with the infiltration of CD8^+^ T cells in GC tissues. Inhibition of ACC can enhance the anti-tumor immune capacity of GC [135]. The expression of the inactive form of ACC (pACC) is decreased in GC, and the level of pACC is also much lower in poorly differentiated GC compared to well-differentiated GC, indicating higher ACC activity in poorly differentiated GC [136]. Patients with higher levels of pACC do have longer survival time [137]. AMPK is an upstream target of ACC, and phosphorylation of AMPK can regulate the activity of ACC. Overexpression of pACC induced by an AMPK activator (metformin) significantly inhibits the growth of GC cells [137,138].

FASN in GC is a prognostic risk factor, similar to other malignancies. Its overexpression is associated with shorter survival. What sets it apart is its ability to control macrophage polarization and play a role in GC tumor formation [139]. Additionally, SCD1 is also upregulated in GC, increasing the proliferation and migration abilities of GC cells, accelerating tumor formation, and exhibiting anti-ferroptosis effects, indicating a poor prognosis. SCD1 also regulates tumor cell stemness through the Hippo/YAP pathway, promoting the occurrence, drug resistance, and metastasis of GC. Knocking down SCD1 can reverse the resistance of GC cells to oxaliplatin [140,141].

As a cell membrane receptor, CD36 regulates fatty acid uptake and immune recognition in various cells [142]. High expression of CD36 is associated with adverse clinical pathological types in GC patients, and upregulation of CD36 indicates a poor prognosis and high metastasis [143,144]. CD36 also acts as a key mediator in the AKT/GSK-3/β-catenin signaling pathway to promote GC metastasis [145]. There are also studies suggesting that fatty acid uptake promotes the transcription and function of CD36 through activation of the NF-kB pathway, leading to cancer metastasis and the formation of a malignant cycle. Considering the important role of CD36 in GC invasion, targeting CD36 may help reduce GC metastasis [146].

In relation to FAO, CPT1A protein is one of the members of the CPT family, significantly elevated in GC cells and associated with clinical pathological classification and poor prognosis of GC. Overexpression of CPT1A activates FAO in GC cells by increasing the NADP/NADPH ratio, promoting proliferation, invasion, and EMT of GC cells [147,148]. CPT1A also inhibits the degradation of lactate dehydrogenase A (LDHA) and promotes GC invasion and proliferation through succinylation of LDHA [149]. Additionally, hypoxia-induced expression of CPT1C has been shown to enhance GC cell proliferation [150,151].

### 3.5. Breast cancer

Proteins involved in fatty acid (FA) synthesis and oxidation play a crucial role in the proliferation, migration, and invasion of breast cancer (BC) cells. However, unlike other cancers, BC exhibits distinct FA metabolism differences among its molecular subtypes (RNBC, RPBC, and TNBC). Based on the upregulation of the leptin-LEPR-JAK-STAT3 pathway, human BC cell lines have shown an increased bioenergetics dependency on FAO [152]. However, compared to other subtypes, TNBC appears to rely more on the uptake and storage of exogenous FAs, while RPBC exhibits a greater degree of upregulation in FAs synthesis and oxidation [153].

ACLY is upregulated in malignant breast tumors and plays a crucial role in the transformation, migration, invasion, and *in vivo* growth of BC cells. Among the subtypes, ACLY expression is highest in the HER2-enriched subtype, while its expression is relatively lower in TNBC [154,155]. ACC activity is associated with the malignant phenotype of BC, with its expression upregulated *in situ* ductal carcinoma and lobular carcinoma, and HER2 overexpression playing a significant role in catalyzing ACC activity [156,157]. Thus, BC cell lines overexpressing HER2 have higher levels of ACC compared to cell lines with lower HER2 expression, and the latter induce ACC expression upon exogenous HER2 stimulation, seemingly regulated through the PI3K/Akt/mTOR pathway [158]. SN is also associated with malignant transformation and poorer prognosis of BC. Upregulation of FASN activity is driven by increased EGF signaling, which also involves the MAPK, PI3K, and SREBP pathways. Additionally, HER2 has been shown to play a role in the regulation of FASN activity [18]. In HER2-positive BC cells, FASN expression increases, and inhibition of HER2 activity with inhibitors leads to decreased FASN expression. Mechanistically, HER2 stimulates the FASN promoter through PI3K activation, and HER2 can directly phosphorylate FASN, thus activating its activity. Inhibiting FASN phosphorylation can reduce the invasiveness of BC cells. This is also one of the main reasons for the increase in FAs synthesis in RPBC [159]. Forcing upregulation of FASN in normal mammary epithelial cells leads to their transformation into malignant cells and increases the expression levels of EGFR and HER2 [160]. Furthermore, the application of FASN inhibitors (such as cerulenin) in BC has been reported to increase apoptosis in HER2-amplified BC cells, suggesting that the effect of FASN inhibitors on HER2 expression could be an effective therapeutic target for BC [161].

In highly proliferative cells, the consumption of FAs as fuel can disrupt membrane formation. Therefore, FAO is prioritized as an energy source for resting cells. Similar to FAs synthesis, FAO is more active in hormone RPBC compared to TNBC. On one hand, carnitine palmitoyltransferase 1 A (CPT1A) expression is higher in RPBC than in TNBC. On the other hand, acyl-CoA oxidase 2 (ACOX2), which is the rate-limiting enzyme for the beta-oxidation of long-chain FAs, is overexpressed in RPBC subtypes. However, the predominant expression of these related proteins in RPBC does not exclude the role of FAO in TNBC. FAO still provides a considerable energy source for TNBC cell lines [162–164].

The expression of proteins involved in lipid droplet formation, such as TNBC, is increased. LDs are subcellular organelles composed of a phospholipid monolayer membrane, primarily consisting of TG and cholesterol esters. Nutritional deficiency and hypoxia can induce the formation of LDs, thereby enhancing the survival of cancer cells [48,165]. LPIN1 is a phosphatidic acid phosphatase that catalyzes the formation of TG. LPIN1 is overexpressed in TNBC and knocking down LPIN1 can inhibit its proliferation and migration. Similarly, this includes PLIN1 and PLA2G4A [54,166]. Lipoprotein lipase (LPL) participates in the uptake of exogenous FAs. Basal-like BC cell lines express significantly higher levels of LPL, and LPL is preferentially expressed in TNBC tissue samples. Another receptor mediating the uptake of exogenous FAs is the very low-density lipoprotein receptor (VLDLR). Compared to normal tissue, the expression of VLDLR protein is upregulated in BC and the upregulation level is positively correlated with lymph node metastasis. Similarly, the expression of VLDLR is higher in the TNBC subtype [167,168]. Overall, different BC subtypes have distinct fatty acid metabolism phenotypes, which deserve further investigation and may provide strategies for the precision treatment of BC patients.

## 4. The significance of targeting fatty acid metabolism in anticancer therapy

**Figure 5.**
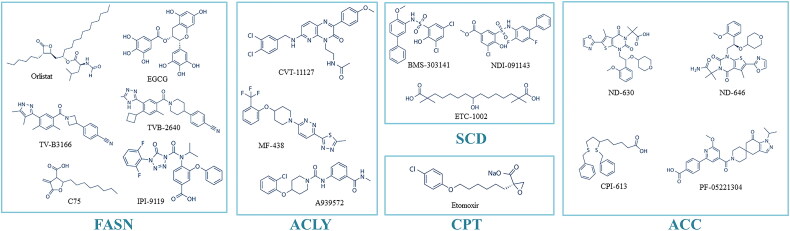
Structural formula of small molecule anticancer drugs targeting FA metabolism.

Given the widespread involvement of FA metabolism in cancer pathogenesis, the development of anticancer therapies targeting FA metabolism reprogramming holds significant clinical significance. Although there have been ongoing developments in drugs targeting FA metabolism, the progress in targeting FA metabolism in cancer therapy has been limited, with only a few drugs entering clinical trials (Table 2). Additionally, specific dietary interventions to enhance the efficacy of existing anticancer treatments have also gained interest. Here, we summarize various drug developments targeting FA metabolism reprogramming and the effectiveness of synergistic and rational exogenous FAs intake in enhancing anticancer efficacy. Figure 5 illustrates the structural formulas of various small molecule drugs targeting fatty acid metabolism (Figure 5).

**Table 2.** Research progress of drugs targeting FA metabolism.

FAs metabolic process	Enzyme target	Drug	Cancer type	Research progress	Trial ID or PMID	References
De novo synthesis	FASN	Cerulenin	Breast caner	Preclinical	36308575	[1]
			Ovarian cancer	Preclinical	25947066	[2]
			Colorectal cancer	Preclinical	26109148	[3]
			Adenocarcinoma lung cancer	Preclinical	28400509	[4]
			Bladder cancer	Preclinical	22554590	[5]
		C75	Gastric cancer	Preclinical	28339092	[6]
			Breast caner	Preclinical	17902053	[7]
			Adenocarcinoma lung cancer	Preclinical	22769244	[8]
			Prostate cancer	Preclinical	22565411	[9]
		Orlistat	Pancreatic cancer	Preclinical	29061815	[10]
			T-cell leukemia	Preclinical	31775585	[11]
			Breast caner	Preclinical	36528070	[12]
			Gliomas	Preclinical	24789255	[13]
			Hepatocellular carcinoma	Preclinical	32045588	[14]
			Ovarian cancer	Preclinical	22581080	[15]
			Prostate cancer	Preclinical	33974005	[16]
		TVB-3166	Ovarian cancer	Preclinical	26425687	[17]
			Breast caner	Preclinical	33893909	[18]
			Colorectal cancer	Preclinical	26425687	[17]
			Non-small cell lung cancer	Preclinical	26425687	[17]
		TVB-2640	Non-small cell lung cancer	Phase II Clinical Trial (ongoing)	NCT03808558	[19]
			Colon cancer	Window Trial	NCT02980029	[19]
			HER2+ Metastatic Breast Cancer	Phase II Clinical Trial (ongoing)	NCT03179904	[19]
	ACC	Soraphen A	Prostate cancer	Preclinical	NCT05743621	[20]
		BAY ACC002	Pancreatic cancer	Preclinical	17804731	[21]
		ND-654	Hepatocellular carcinoma	Preclinical	27750213	[22]
		ND-646	Non-small cell lung cancer	Preclinical	30244972	[23]
	ACLY	SB-204990	Thyroid cancer	Preclinical	27643638	[24]
		NDI-091143	Thyroid cancer	Preclinical	35761130	[24]
		BMS-303141	Hepatocellular carcinoma	Preclinical	35761130	[25]
		Hydroxycitrate	Chronic Myelogenous Leukemia	Preclinical	33393219	[26]
			Breast caner	Preclinical	35807850	[27]
	SCD	A939572	Clear cell renal cell carcinoma	Preclinical	36401980	[28]
			Pancreatic cancer	Preclinical	23633458	[29]
			Non-small cell lung cancer	Preclinical	33565799	[30]
			Bladder cancer	Preclinical	35110426	[31]
			Ovarian cancer	Preclinical	30592142	[32]
		CAY10566	Breast caner	Preclinical	31270077	[33]
			Hepatocellular carcinoma	Preclinical	25013434	[34]
			Ovarian cancer	Preclinical	27784428	[32]
		MF-438	Esophageal squamous cell carcinoma	Preclinical	31270077	[35]
			Ovarian cancer	Preclinical	35859734	[32]
			Lung cancer	Preclinical	31270077	[36]
			Breast caner	Preclinical	28797843	[37]
		CVT-11127	Lung cancer	Preclinical	31642233	[38]
FAO	CPT	Etomoxir	Prostate cancer	Preclinical	22946088	[39]
			Leukemia	Preclinical	25122071	[40]
			Metastatic ovarian cancer	Preclinical	20038799	[41]
		ST1326	Lymphoma	Preclinical	35633555	[42]
			Leukemia	Preclinical	23486551	[43]
		Perhexiline	Pancreatic ductal adenocarcinoma	Preclinical	26276667	[44]
			Chronic lymphocytic leukemia	Preclinical	37305702	[45]
			Hepatocellular carcinoma	Preclinical	27065330	[46]
			Breast caner	Preclinical	29795111	[47]
FAs exogenous uptake	FABP	BMS3094013	Multiple myeloma cell	Preclinical	25849870	[48]
		SBFI-26	Multiple myeloma cell	Preclinical	36880649	[48]
			Breast caner	Preclinical	36880649	[49]

### 4.1. Targeting FASN

As a key enzyme in FAs synthesis, FASN was the first to be targeted by drugs aimed at FA metabolism. The first-generation FASN inhibitors include C75, orlistat, epigallocatechin gallate (EGCG), and cerulenin, which were initially found to significantly reduce the growth of tumor xenografts, induce cell cycle arrest, and suppress the transcription of oncogenes. Additionally, they sensitize BC cell lines to chemotherapy-induced cell death by reducing the synthesis of saturated FAs [169,170]. However, despite successful results in *in vitro* experiments, early FASN inhibitors did not demonstrate ideal efficacy *in vivo*, often due to inadequate drug distribution and specificity. For example, the expression levels of FASN in proliferative non-malignant ovarian epithelial cells and tubal secretory epithelial cells are comparable to that of ovarian cancer cell lines, and inhibitors such as C75 and G28UCM exhibit similar sensitivity against the three cell lines. Fortunately, in normal cells, C75 only slows down the cell cycle without inducing apoptosis, allowing their survival despite growth inhibition caused by FASN blockade, while malignant cells are eradicated [171]. At the same time, early FASN inhibitors also caused varying degrees of systemic side effects, including significant weight loss and anorexia. Targeting FASN with C75 in mice leads to pronounced appetite suppression and weight loss, whereas EGCG, due to its lack of significant impact on CPT-1 activity, often does not cause weight loss while inhibiting the growth of adenocarcinoma- and lung carcinoma-derived xenografts [172]. IPI-9119 is an irreversible inhibitor of the thioesterase domain targeting FASN. However, IPI-9119 does not exert significant antiproliferative effects on various tumor cell lines *in vitro*, suggesting that inhibiting FASN alone may not be sufficient to impact cancer cell proliferation and tumor growth. Similar to IPI-9119, orlistat, a simplified version of lipstatin, has been approved for combating obesity by blocking the absorption of FAs in the gastrointestinal tract. It demonstrates potential antitumor properties by covalently binding to FASN. However, problems with orlistat’s absorption rate have hampered progress in clinical trials [173,174].

Recently, novel FASN inhibitors (TVB-3166 and TVB-2640) have shown significant anticancer potential and lower systemic toxicity in BC, ovarian cancer, non-small cell lung cancer, and colorectal cancer. In early clinical trials, they demonstrated higher specificity and limited off-target effects, which may be attributed to the fact that TVB-3166 and TVB-2640 do not indirectly activate CPT1 in peripheral tissues [175–177]. The anticancer effects of TVB are associated with a significant reduction in the adenine nucleotide pool and changes in lipid composition, particularly the increase in lactosylceramide and sphingomyelin, which are sensitive to FASN inhibition. Moreover, Akt, Erk2/1, and AMPK are the major carcinogenic pathways involved in TVB-mediated effects [178]. Compared to early FASN inhibitors, TVB-induced adverse reactions are mostly mild, and all adverse events can gradually recover after dose interruption [179].

### 4.2. Targeting ACC

Another approach to inhibiting FAs synthesis is through the inhibition of ACC. Unlike FASN inhibition, this strategy may result in a reduction in malonyl-CoA, thereby stimulating FAO. ACC plays a critical regulatory role in both FAs synthesis and oxidation pathways, making it an effective target for various metabolic disorders. Currently, effective ACC inhibitors include Soraphen A, ND-630, ND-646, and PF-05221304 [180,181]. Soraphen A inhibits ACC1/2, leading to a decrease in cellular levels of acetyl-CoA and stalling of FAs chain elongation, ultimately resulting in cell death. However, its poor specificity and pharmacokinetic properties limit its clinical application [158,182]. ND-630 and PF-05221304 have shown preliminary efficacy in phase I studies targeting hepatocellular carcinoma, with mild adverse reactions meeting further requirements for anticancer treatment [183]. Additionally, AMPK plays an important role in the phosphorylation imbalance of ACC, and some drugs targeting AMPK can also affect ACC activity. For example, metformin effectively inhibits ACC by activating AMPK, demonstrating certain anticancer effects [184,185]. CPI-613 (Devimistat), a novel fatty acid analog that inhibits mitochondrial metabolism, enhances apoptosis in pancreatic cancer cells by inhibiting the AMPK-ACC signaling pathway [186].

### 4.3. Targeting ACLY

ACLY (ATP citrate lyase) catalyzes the conversion of citrate to acetyl-CoA, which is essential for FAs synthesis [187,188]. Increased expression and activity of ACLY have been observed in various cancers including glioblastoma, colorectal cancer, BC, and hepatocellular carcinoma, suggesting that targeting ACLY may be an effective approach for anticancer therapy. In thyroid cancer cell lines, ACLY inhibitors have been found to dose-dependently and time-dependently inhibit monolayer cell growth and clonogenic ability, as well as synergistically enhance the cytotoxicity of sorafenib [189]. The anticancer effects of ACLY inhibitors, such as SB-204990 and hydroxy citrate, are particularly prominent in highly glycolytic cells, possibly because the main source of acetyl-CoA generated by ACLY is derived from glucose-derived citrate salts [190]. However, the clinical feasibility of these compounds is not optimistic due to their poor pharmacokinetic properties. In contrast, a novel allosteric ACLY inhibitor, NDI-091143, shows high activity and holds promise for guiding anticancer treatments [191].

In addition to blocking FAs synthesis by inhibiting the conversion of citrate to acetyl-CoA, ACLY inhibitors also play an important role in the signal network of energy stress response. Based on this, another approach in cancer therapy is to combine ACLY inhibitors with AMPK activators [192]. For example, ACLY inhibition (BMS-303141) in CRPC cells promotes energy stress and AMPK activation, disrupting the endoplasmic reticulum and energy balance of CRPC cells. This further inhibits proliferation, promotes apoptosis, and sensitizes the cells to androgen receptor inhibitors. In this context, exogenous FAs can restore the phenotype [192]. ACLY inhibitors currently used to treat hypercholesterolemia, such as ETC-1002, are also dual activators of AMPK. Evaluating the effectiveness and safety of these drugs in cancer treatment is of great interest [193]. Especially in terms of drug resistance, ACLY inhibitors enhance the pharmacological activity of cisplatin against ovarian cancer cell lines by down-regulating the PI3K-AKT pathway and up-regulating the AMPK-ROS pathway [194].

### 4.4. Targeting SCD

SCD is a key enzyme involved in the synthesis of MUFAs. SFAs are converted into MUFAs through SCD. SCD represents a new generation of targeted therapy for cancer treatment, as inhibiting SCD can result in three effects: accumulation of free fatty acids (FFAs), increased FAO, and reduced fat storage. The conversion between SFAs and MUFAs regulated by SCD profoundly affects the survival of tumor cells and cancer progression [195].

Some small molecule SCD inhibitors have been found to have obvious anti-tumor effects, including BZ36, A939572, CAY10566, MF-438 and CVT-11127, etc. These drugs have shown great potential to suppress cancer in colorectal cancer, renal cell carcinoma, lung cancer, BC and other cancers [114,196–198]. A939572 is a small molecule drug that specifically targets SCD1 and has been shown to inhibit ccRCC proliferation in a dose-dependent manner. In another study, A939572 inhibits migration and invasion in liver cancer and enhances sorafenib drug sensitivity by regulating endoplasmic reticulum stress [199]. The anticancer effects of A939572 have also been observed in pancreatic cancer, bladder cancer, non-small cell lung cancer, and BC [200–203]. Another SCD inhibitor, BZ36, interferes with de novo lipogenesis and carcinogenic signaling in a prostate cancer mouse model, leading to inhibition of tumor growth [101]. In lung cancer cells, inhibition of SCD activity by CVT-11127 impairs phosphorylation of EGFR, resulting in inactivation of downstream targets Akt and ERK, effectively controlling metabolism, proliferation, and survival of tumor cells [204]. Another study also demonstrated that the SCD inhibitor CVT-11127 inhibits SCD activity in human lung cancer cells, inhibiting cancer cell proliferation by blocking cell cycle progression and triggering programmed cell death [197].

Furthermore, ferroptosis is an iron-dependent programmed cell death pathway regulated by LPO, and activation of ferroptosis can significantly inhibit the growth of tumor cells. SCD inhibitors such as MF-1, A939572, and CAY438 inhibit the formation of MUFAs, enhancing the anti-tumor effects of ferroptosis inducer FER-1 in ovarian cancer cell lines and mouse xenograft models. Subsequent studies have also found that combination therapy with death-inducing drugs (such as sorafenib and cisplatin) and SCD blockade may become a new strategy for cancer treatment [73]. In liver cancer cell lines, sorafenib reduces the expression of oncoprotein hepatitis B X-interacting protein (HBXIP), thereby inhibiting SCD activation induced by the transcription factor ZNF263, leading to the accumulation of free FAs and the occurrence of ferroptosis [205].

However, not all cancers exhibit sensitivity to SCD inhibition, as some tumor cells rely on compensatory desaturation pathways that utilize FADS2 to generate sapienate from palmitate esters. Especially in liver cancer and lung cancer, sapienate plays a vital role in tumor cell membrane synthesis. Therefore, for these types of cancer, only the combination therapy of SCD inhibitors and FADS2 inhibitors can block all compensatory pathways for tumor cells to acquire desaturated FAs, leading to impaired proliferation of tumor cells both *in vitro* and *in vivo* [71].

While SCD expression is generally higher in almost all human cancers compared to normal tissues, there are exceptions. For example, lower SCD expression has been observed in glioblastoma, and individual patients with liver cancer and colon cancer can exhibit variable levels of SCD expression. In these cases, the determination of SCD expression levels will be a crucial factor in selecting SCD inhibitors for treatment [206].

### 4.5. Targeting CPT

CPT is a key rate-limiting enzyme in the process of FAO. Its effects on different tissues or organs are complex. The deficiency or excessive activation of CPT can disrupt the immune homeostasis of the body by causing energy metabolism disorders and inflammation-induced oxidative damage. Therefore, targeting CPT may become a new approach for treating diseases [207,208].

In cancer treatment, small molecule CPT inhibitors such as etomoxir and ST1326 have shown promising anti-cancer activity in FAO-dependent cancers, such as prostate cancer and leukemia. Inhibiting CPT reduces the proliferative capacity of tumor cells by blocking FAO, inducing inactivation of AKT kinase, and inhibiting caspase-3 activation, making them more sensitive to apoptotic signals [209,210]. Etomoxir also enhances the sensitivity of various cancers to chemotherapy, including metastatic BC, colon cancer, leukemia, and nasopharyngeal cancer [45,210–212]. However, the toxic side effects of etomoxir should not be overlooked, including increased vascular permeability and hepatotoxicity. Prolonged use of etomoxir can cause cardiac hypertrophy by promoting oxidative stress and activating the NF-kB signaling pathway. Therefore, the development of etomoxir for cancer therapy is still in the preclinical research stage [213–215]. In contrast, liver damage caused by ST1326 is generally reversible, and it exhibits advantageous anti-cancer effects in lymphoma and leukemia by causing mitochondrial damage and cell cycle arrest by blocking the oxidation of long-chain and short-chain FAs [216–219]. In leukemia cells with high levels of CPT1 and CPT2 expression, the anti-anginal drug perhexiline inhibits FAs transport into mitochondria by inhibiting CPT, leading to depletion of phospholipids in mitochondrial membranes and impairing mitochondrial integrity, resulting in cell death in CLL [220].

### 4.6. Fatty acid metabolism and metabolic correlation of cancer treatment resistance

Abnormal fatty acid metabolism of tumor cells plays a crucial role in regulating immune response and chemical cell damage, and can influence the efficacy of anticancer therapy. ACLY is upregulated or activated in many cancers. Inhibition of ACLY leads to PUFA peroxidation and mitochondrial damage, thereby triggering mitochondrial DNA leakage to activate cGAS-STING innate immune pathway, which up-regulates the expression of PD-L1 immune checkpoint in cancer cells. Inhibition of ACLY overcomes resistance to PD-L1 therapy in HCC mice in a Cgas-dependent manner [221]. FAO can enhance chemotherapy resistance of GC, which is related to mesenchymal stem cells (MSC). Mechanistically, MSC induces LncRNA histocompatibility leukocyte antigen complex P5 (HCP5), HCP5 promotes FAO in GC cells, thereby promoting dryness and chemotherapy resistance in GC [222]. By binding with HIF1α, FASN promotes HIF1α nuclear translocation, inhibits HIF1α ubiquitination and proteasome degradation, and then enhances the transcription of carrier family 7 member 11 (SLC7A11), enhancing the iron death resistance of hepatocellular carcinoma cells induced by sorafenib [223]. Adipocyte derived exosome microsomal triglyceride transfer protein (MTTP)/proline-rich acidic protein 1 (PRAP1) complex inhibited the expression of zinc finger E-box binding homeobox 1, upregulation of glutathione peroxidase 4 and xCT, This results in a decrease in the proportion of PUFAs and lipid ROS levels, which reduces the iron death sensitivity of CRC and promotes its chemotherapy resistance to oxaliplatin [224]. Mitochondrial fatty acid β oxidation promotes chemotherapy resistance in breast CSC. CD96 originating from immune cells was found to enhance this effect through the CD155-CD96-Src-Stat3-Opa1 pathway, while inhibition of cancer cell CD96 enhanced chemotherapy response in BC tumor cells [225]. FABP4 is a lipid chaperone protein, and Mukherjee et al. found that knockdown of FABP4 led to increased levels of 5-hydroxymethylcytosine in DNA, down-regulating a genetic signature associated with ovarian cancer metastasis, which reduced tumor load in mouse models of ovarian cancer. It also increases the sensitivity of cancer cells to carboplatin therapy *in vitro* and *in vivo* [226]. Changes in the composition of FAs can also affect the chemotherapy efficacy of cancer, and studies have found that changing the PUFAs and MUFAs ratios of mouse cell membranes by fasting will isolate the therapeutic effects of oxaliplatin and adriamycin [227]. Another lipidomics analysis of CRC showed that the inactivation of SCD1 in HCT-116 and DLD-1 cells led to an imbalance in the ratio of SFAs to MUFAs on the cell membrane, which reduced cell membrane fluidity and increased the resistance of CRC tissue to 5-FU [228].

Fatty acid metabolism may affect cancer treatment by affecting the metabolism of other substances. Increased FAs uptake was found to be associated with cancer cell resistance to cisplatin, and increased FAs uptake promoted cancer cell survival under cisplatin induced oxidative stress by enhancing beta-oxidation. Furthermore, increased FAs uptake by cisplatin-resistant cells was accompanied by decreased glucose uptake and lipogenesis, suggesting that glucose was reprogrammed into FA-dependent metabolism [229]. Venettok combined with azacitidine (ven/aza) is an effective regimen for the treatment of AML, and the up-regulation of FAO enhances the resistance of ven/aza. Studies have shown that ven/aza acts on AML stem cells mainly by inhibiting amino acid metabolism, and FAO eliminates their need for amino acid metabolism, thereby reducing ven/aza therapeutic sensitivity [230].

Related studies have also found that the accumulation of LDs is closely related to cancer chemotherapy resistance. For example, LDs accumulates in liver cancer cells in the form of aldoketoreductase 1C3(AKR1C3) dependent, which mitigated mitochondrial lipid toxicity and dysfunction induced by sorafenib. Inhibition of AKR1C3 activity can induce LDs catabolism, leading to mitochondrial division and apoptosis in sorafenib resistant hepatocellular carcinoma (HCC) [231]. Another study found that LDs accumulation makes metastatic cancer cells resistant to paclitaxel therapy, that by mechanism, LDs accumulation reduces ROS levels, thereby maintaining endoplasmic reticulum (ER) homeostasis in paclitaxel-resistant cells, and that this LD accumulation is associated with high expression of the oncogenic transcription factor FOXM1 [232] (Figure 2).

## 5. Dietary structure influences the efficacy of fatty acid metabolism in cancer

It is well known that diet is closely related to the occurrence of certain diseases, and the types and amounts of exogenous FAs consumed through diet can impact the development and progression of tumors. For example, high-fat diets have been shown to provide opportunities for the development of intestinal tumors by activating FAO [233]. As early as 1998, the impact of dietary fat composition on the growth and metastasis of BC was discovered. A diet containing 9.5% eicosapentaenoic acid and 0.5% linoleic acid reduced the growth and metastasis of tumor cells, while a diet containing 10% linoleic acid enhanced the malignant proliferation of cancer cells [234].

Essential fatty acids in the human body include linoleic acid, alpha-linolenic acid, and docosahexaenoic acid, all of which are PUFA. Linoleic acid, dihomogamma-linolenic acid (DGLA), and others belong to the omega-6 FAs category, while alpha-linolenic acid and docosahexaenoic acid belong to the omega-3 FAs category. Omega-3 FAs are mainly found in marine animals and plants (fish, shrimp, seaweed, etc.), while foods rich in omega-6 FAs are abundant and include corn, soybean, pork, beef, etc. However, research shows that a high intake of omega-6 FAs increases the risk of prostate and BC, whereas a diet rich in omega-3 FAs helps improve inflammation and inhibit the growth of breast and colorectal cancer [235–238]. There are also reports that excessive intake of omega-3 FAs can suppress host immunity, which is unfavorable for cancer resistance [239]. In addition, omega-3 also selectively induced iron death of cancer cells in an acidic environment, and related studies compared it to a diet rich in MUFAs, a diet rich in omega-3 long FAs significantly delayed tumor growth in mice, and iron death inducers enhanced this phenomenon [68]. Therefore, determining the ideal ratio of omega-3 FAs to omega-6 FAs in food is crucial for disease dietary management.

In addition, palmitic acid (PA) is a common fatty acid in the human diet and acts as a signaling molecule in many disease progressions, including metabolic syndrome, cardiovascular disease, neurodegenerative diseases, and cancer. A diet rich in PA has unique oncogenic characteristics in promoting colorectal cancer growth, which is based on the activation of the β-adrenergic signaling pathway [240]. In terms of cancer metastasis, dietary PA has been found to promote the metastasis of oral cancer and melanoma in mice, and this PA-induced prometastatic memory requires the involvement of the FATP CD36 [241,242]. Another study has also shown that a high-PA diet promotes TLR4-induced IFN-β expression, leading to lung metastasis in melanoma [243].

Ketogenic diet (KD) may influence tumor progression by regulating lipid metabolism.While KD damages tumor SCD activity, the increased lipid availability driven by KD maintains the ratio of unsaturated fatty acids to SFAs in the tumor. This situation can be avoided by changing KD fat composition to slow down tumor growth [244]. Ketosome beta-hydroxybutyrate (BHB) inhibits the proliferation and growth of CRC by regulating the homologous domain protein Hopx on cancer cells, which requires the surface receptor Hcar to induce Hopx expression and inhibit the proliferation of intestinal epithelial cells [245]. The ketogenic diet also affects the immunotherapeutic effects of cancer, with 3-hydroxybutyrate (3HB) preventing the up-regulation of immune checkpoint blocking of PD-L1 on myeloid cells in mouse models of melanoma, kidney cancer, and non-small cell lung cancer, while favoring the expansion of CXCR3 + T cells, thereby enhancing the anti-cancer effects of PD-1 blocking [246]. However, excessive ketogenic diet may aggravate tumor development. In a study evaluating the effects of an unrestricted ketogenic diet on tumor growth in EOC, KD showed significant enrichment in PPAR signaling and fatty acid metabolism pathways, suggesting that an unrestricted KD diet promotes tumor progression [247].

## 6. Conclusion

This article discusses extensive research highlighting the complex FA metabolic reprogramming in tumor cells. This includes the highly activated de novo synthesis pathway in tumor cells to meet their intense biosynthetic needs and support their proliferation and metastasis energy demands through active FAO. Exogenous uptake also serves as an important source of FAs for tumor cells. With changes in FA metabolism, certain oncogenic signaling pathways, such as the PI3K-AKT-mTOR pathway, are also rewired, often leading to remodeling of the tumor microenvironment. Ferroptosis is a novel non-apoptotic programmed cell death process, which is different from other identified programmed cell death and has unique morphological and metabolism characteristics. We conclude that abnormal FA metabolism in tumor cells can induce alterations in ferroptosis signaling, mainly abnormalities in substrates involved in LPO, which show great potential in anti-cancer therapies targeting programmed cell death.

Furthermore, we demonstrate the differential FA metabolic reprogramming in different cancer types and subtypes. The most distinct characteristic is that TNBC relies more on exogenous FA uptake and storage compared to RPBC, which exhibits more active FAs synthesis and oxidation. These differences are determined by variations in enzyme activities and induction by various regulatory factors (such as HER2) in the FA metabolic pathway. Close attention to the dynamic changes in the FA metabolic pathway and characterization of the FA metabolic reprogramming in different cancer types helps us gain further insight into the intrinsic mechanisms of human cancer and explore the patterns of cancer development.

We also focus on discussing specific enzyme-targeted anti-tumor inhibitors in the FA metabolic pathway, with a particular emphasis on targeting FASN and SCD, which have gained broad attention. However, certain small molecule inhibitors face obstacles in clinical development due to off-target effects and severe systemic toxicity. Additionally, although targeting FA metabolism is a promising therapeutic strategy, not all tumor cells exhibit the same metabolic patterns, as they are often activated by different enzymes or receptors. Therefore, determining the metabolic susceptibility of tumor cell populations and implementing personalized treatment plans are crucial.

In summary, in view of the complexity of cancer metabolic networks, targeting FA metabolism is a very promising direction to conquer cancer and provide efficient targeted therapy for cancer patients.

## Data Availability

Data sharing is not applicable to this article as no new data were created or analyzed in this study.
